# Supplementary material for: A molecular T-pentomino for separating BTEX hydrocarbons
Source: Nat Commun. 2024 Mar 8;15:2121. doi: 10.1038/s41467-024-45542-2 (PMC10924097; doi:10.1038/s41467-024-45542-2)
Supplement: Supplementary file 1 — Supplementary information [file 41467_2024_45542_MOESM1_ESM.pdf]

Supplementary Information

A molecular T-pentomino for separating BTEX hydrocarbons

Christopher J. Hartwick, Eric W. Reinheimer, Leonard R. MacGillivray*

Department of Chemistry, University of Iowa, Iowa City, IA, 52242

Supplementary Methods

U-shaped bipyridine synthesis.

All materials were obtained from commercial sources and used as received unless indicated. Dibromonaphthalene and palladium bis-triphenylphosphine dichloride were purchased from A2B Chem. Catechol, 4-vinyl pyridine, dichloromethane (99%) toluene (99%), and triethylamine were all purchased from Sigma- Aldrich. Xylenes (95%) and ethylbenzene (99%) were purchased from Acros. Phenylboronic acid was purchased from Oakwood chemicals.

DEPN. To a 150ml round bottom flask containing a degassed (argon, 10 minutes) solution of 50 ml acetonitrile and 75 ml of triethylamine, 1,8-dibromonaphthalene (20 mMols) was added and stirred until dissolved. Palladium bis-triphenylphosphine dichloride (10 mol %, 0.2 mMols) was added while under argon. After filtering through silica gel to remove polymerized material, 4-vinyl pyridine was added to the reaction mixture and stirred at reflux for 72 hours. After TLC (ethyl acetate) showed no bromoarene remaining, the reaction mixture was cooled and rotary evaporated until solid remained. This material was eluted through a flash column using ethyl acetate until the solution was clear instead of yellow. The resulting liquid was again rotary evaporated to yield a solid. Recrystallization from ethyl acetate granted shiny, crystalline material after washing with acetonitrile. Yield: 60.3%, White-yellow crystals. ^{1}H NMR data agree with reported values.[1]

Assembly formation

DBP-DEPN

DEPN (0.9 mmols), phenyboronic acid (1.82 mmols), and catechol (1.82 mmols) are first ground with drops of dichloromethane/toluene (1:1 solution). The resulting powder was scraped from the grinding apparatus and placed into a 30 ml scintillation vial. The mortar-and-pestle were rinsed with another 4.0 ml (2 x 2.0 ml) of solution dropwise and ground to agitate any attached material, which was then added to the vial. The resulting suspension was heated until the powder had dissolved. Upon cooling, translucent bright

yellow crystals appeared within 20 minutes in near quantitative yield. The resulting powder was oven dried at 80 ^{o}C for a minimum of 4 hours. Use of dichloromethane is not necessary for crystallization to proceed, however it allows for ease of solids separation.

Crystallization Experiments

Aromatic guest free solid was grown in open air from dichloromethane, resulting in a single molecule inclusion species. For each crystallization with a BTEX hydrocarbon, a scintillation vial containing ground **DBP-DEPN** (0.06-0.08mmols) was filled with 2.0 ml of BTEX hydrocarbon, gently heated, and allow to cool to room temperature.

Competitive crystallization experiments were performed in the same manner as above but with increased **DBP-DEPN** (0.13-0.16 mmols). Crystallization of **DBP-DEPN** with a 1:1 w/w mixture of thiophene and benzene (2.0 ml) following heating resulted in crystals containing only benzene, as determined by ^{1}H NMR spectroscopy. PXRD data matched the host material. The procedure was repeated with both a 1:1 w/w mixture of ethylbenzene and styrene (2.0 ml) as well as a standard mixture (3.0 ml) of commercial xylenes (~2(*m)*:1(*p)*:1*(o)*:1(EB)).

Additionally, competition between guests was undertaken with benzene, toluene, and ethylbenzene being mixed equal parts by mass (0.1mmols of each, equimolar ratio) with 1:1 ratios for competition between benzene and toluene, benzene and ethylbenzene, toluene and ethylbenzene, and a 1:1:1 ratio of the three together. Crystallizations occurred within 5 minutes after a 1:20 mass ratio of host to guest was added to the respective solution in a 30ml scintillation vial and gently heated to ensure dissolution. The crystalline product was removed, and excess solution was allowed to evaporate for 20 minutes.

Aromatic Guest-Free (CH_2Cl_2 solvate)

^{1}H NMR (400 MHz, $CDCl_3$) δ 8.56 (d, J = 5.5 Hz, 4H, H_f), 8.15 (d, J= 4, 16Hz, 2 H, H_h), 7.99 (d, J = 7.4 Hz,4H,H_j), 7.75 – 7.73 (m, 4H,H_c), 7.72 (d, J = 3.3 Hz, 2H,H_k),7.49 (d, J = 5.5 Hz, 4H, H_g), 7.37 (m, J = 3.6 Hz, 4H, H_d), 6.98 (d, J= 4, 16Hz, 2 H, H_h), 6.88 – 6.87 (m, 2H, H_e), 6.86 (d, J = 2.0 Hz, 4H,H_b), 6.75 (d, J = 2.0 Hz, 4H,H_a), 5.36 (s,1H).

Solvate characterization performed using a Exeter Analytical CE-440 addressing carbon, hydrogen, and nitrogen. Analysis calculated for $C_{48}H_{36}N_2O_4$, C: 79.36 H:4.99 N:3.86. Found: C: 73.44 H: 4.77 N:2.83

Aromatic Guest-Free (Dried in oven)

^{1}H NMR (400 MHz, $CDCl_3$) δ 8.49 (d, J = 5.5 Hz, 4H, H_f), 8.16 (d, J= 4, 16Hz, 2 H, H_h), 7.89 (dd, 4H,H_c), 7.65 (d, 2H,H_J), 7.52 (t, 2H, H_k), 7.45 (m-overlapped, 4H, H_d), 7.43 (m-overlapped, 2H,H_L), 7.41 (m-overlapped, 2H, H_e), 7.31 (d, J = 5.5 Hz, 4H, H_g), 7.17 (m, J = 1.9 Hz, 4H, H_b), 6.99 (m, J = 1.9 Hz, 4H, H_a), 6.92 (d, J= 4, 16Hz, 2 H, H_i).

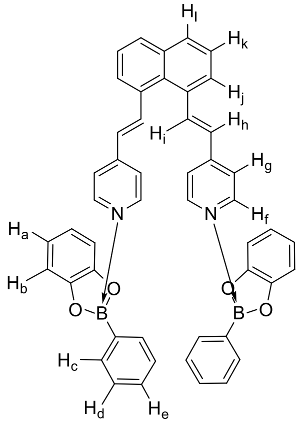

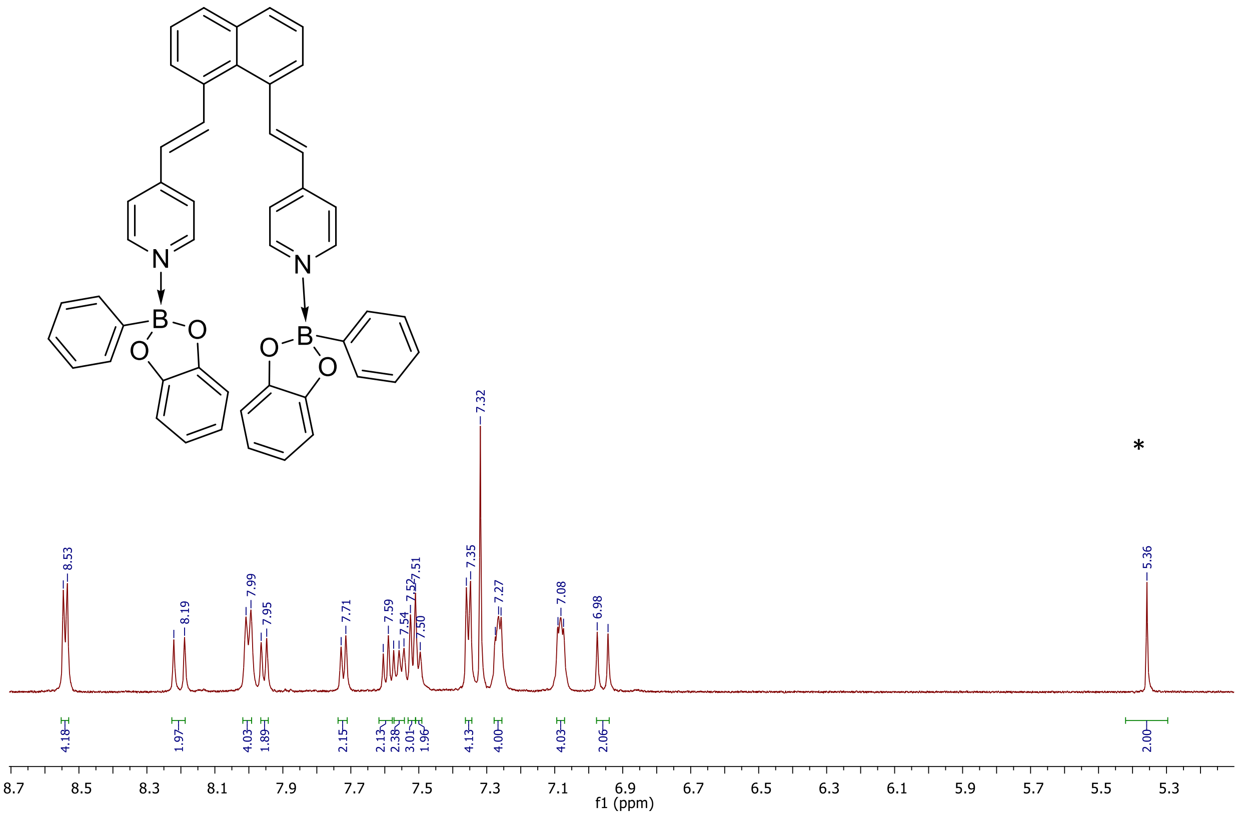

Supplementary Figure 1. ^{1}H NMR spectrum of assembly in $CDCl_3$, grown from CH_2Cl_2 (black, 1 included molecule, 5.36 ppm).

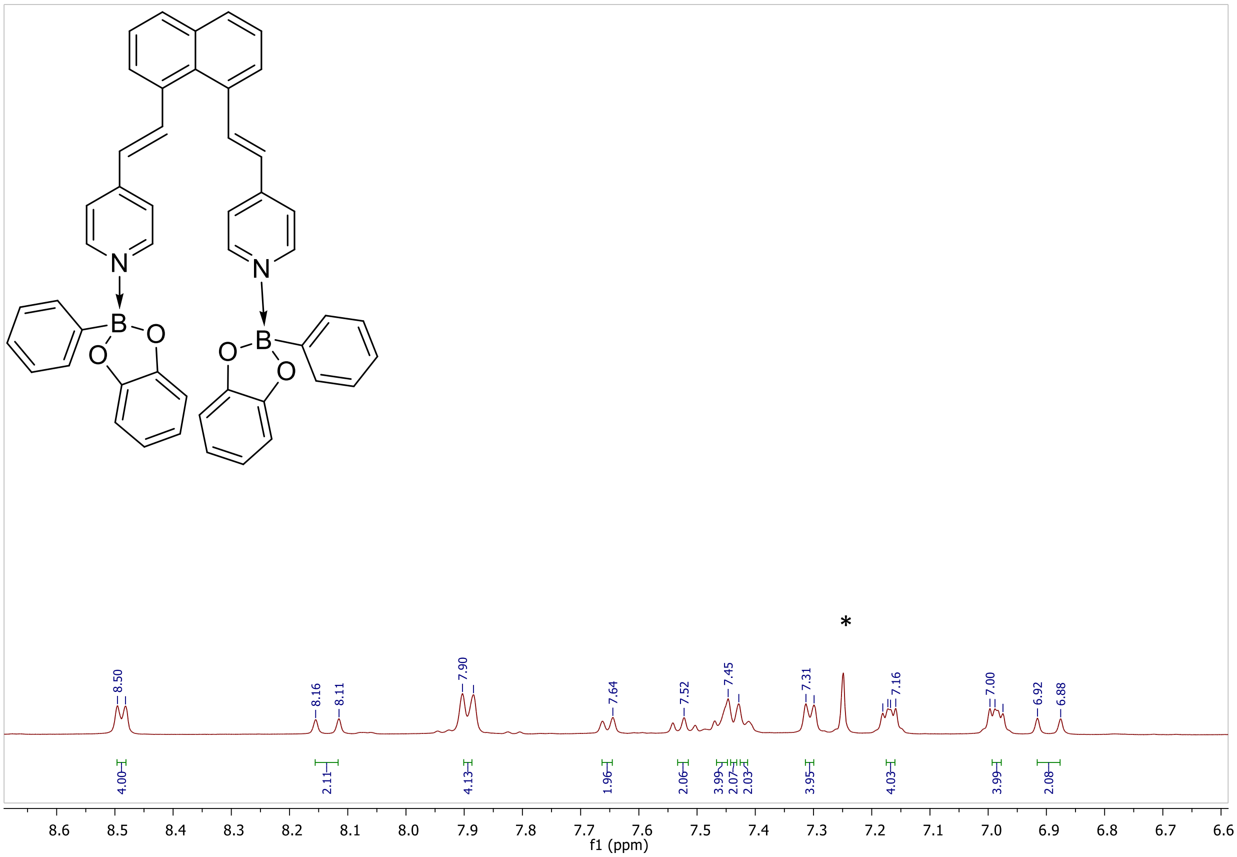

Supplementary Figure 2. ^{1}H NMR spectrum of assembly in $CDCl_3$. Solvent residue: chloroform (black).

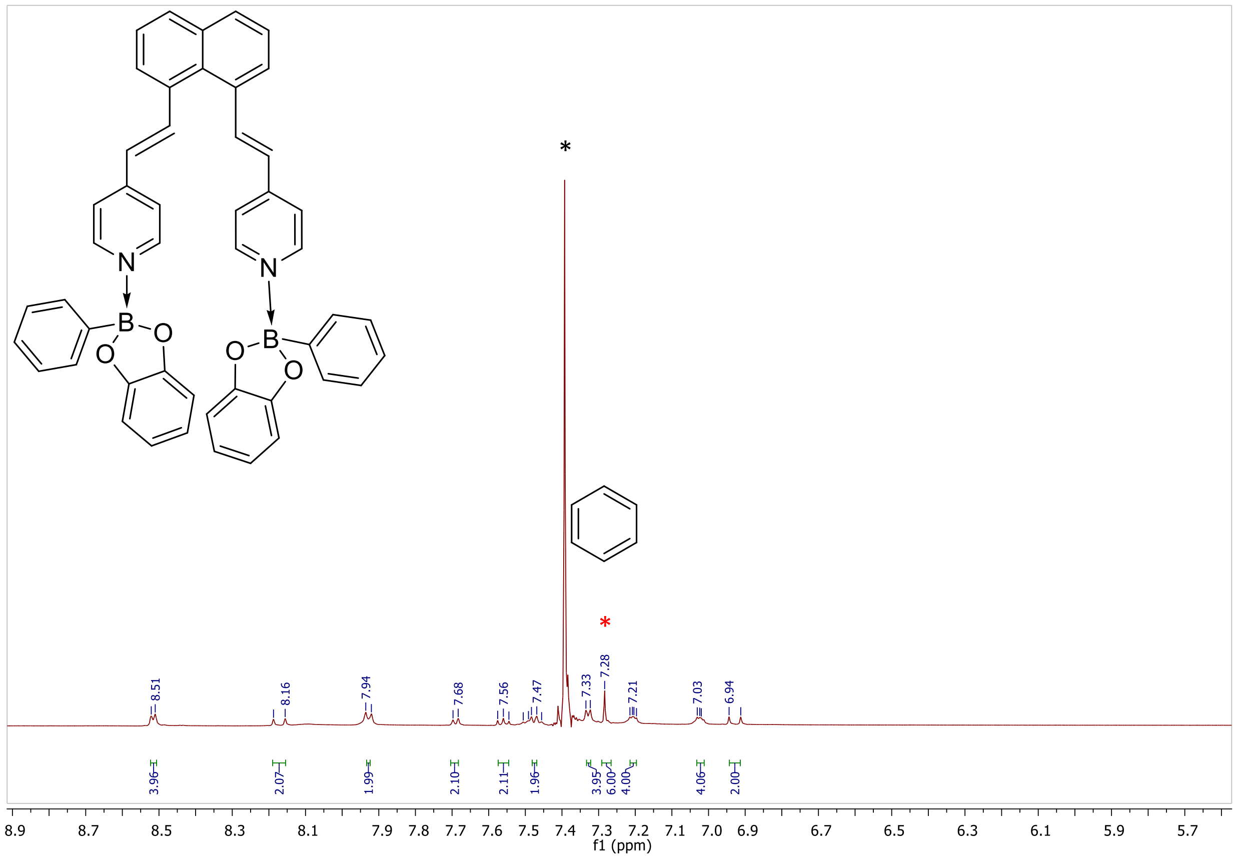

Supplementary Figure 3. ^{1}H NMR spectrum of **DBP-DEPN**·benzene. Solvent inclusion: benzene (red), solvent residue: chloroform (black).

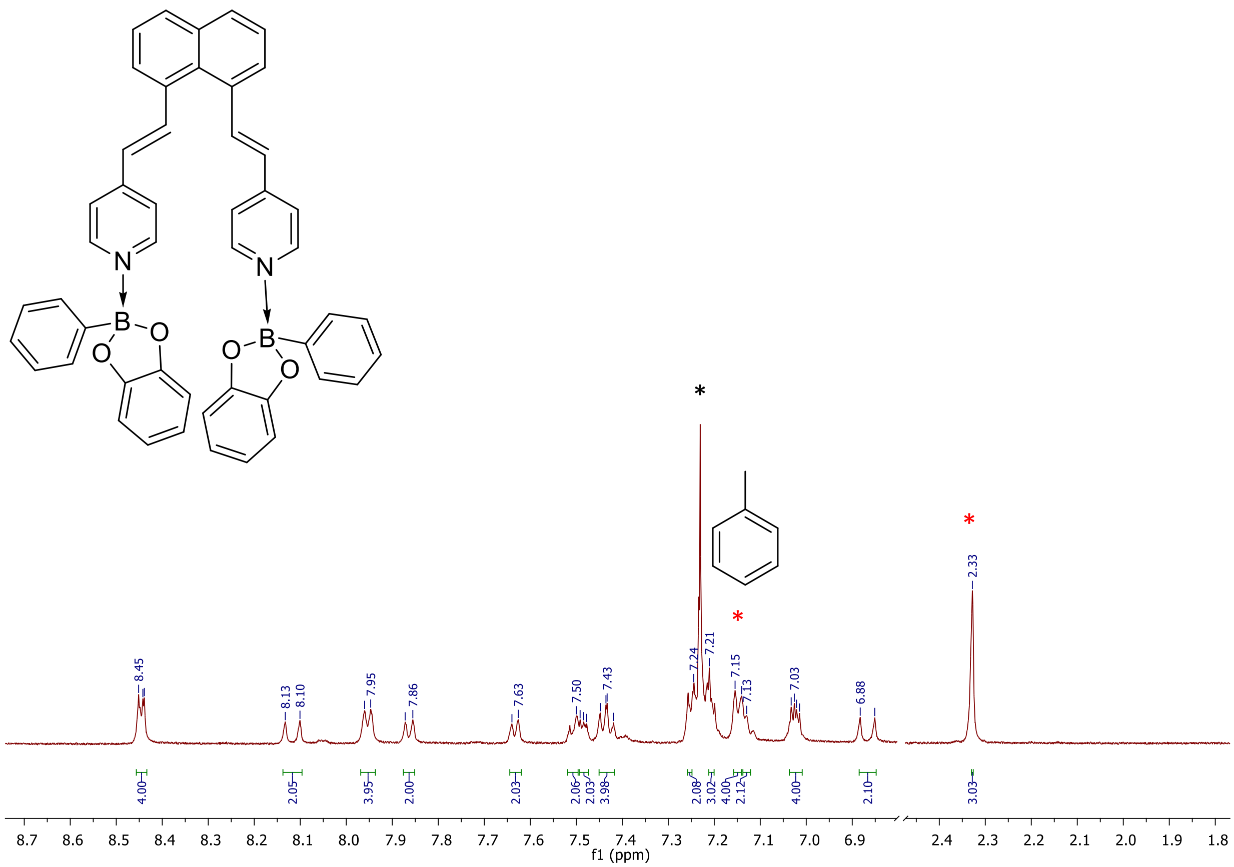

Supplementary Figure 4. ^{1}H NMR spectrum of **DBP-DEPN**·toluene. Solvent inclusion: toluene (red), solvent residue: chloroform (black).

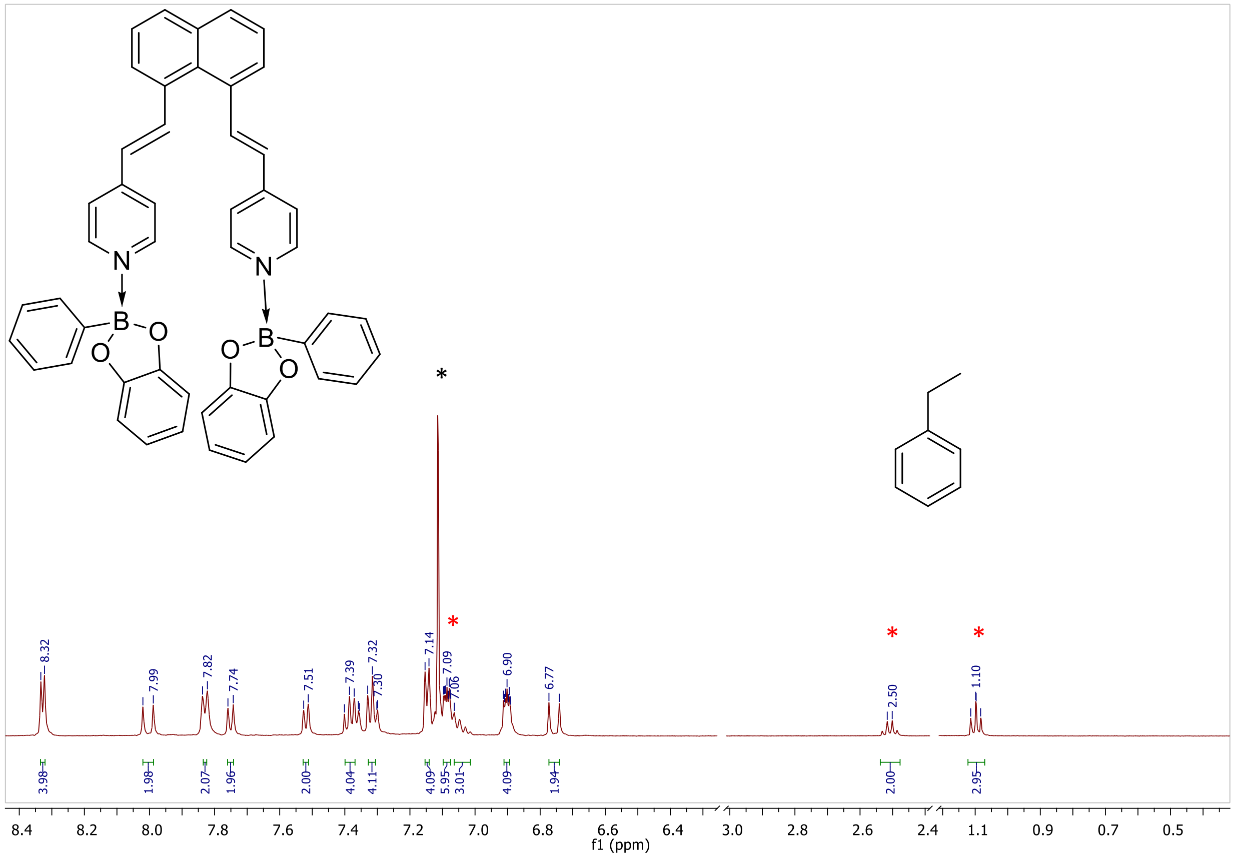

Supplementary Figure 5. ^{1}H NMR spectrum of **DBP-DEPN**·ethylbenzene. Solvent inclusion: ethylbenzene (red), solvent residue: chloroform (black).

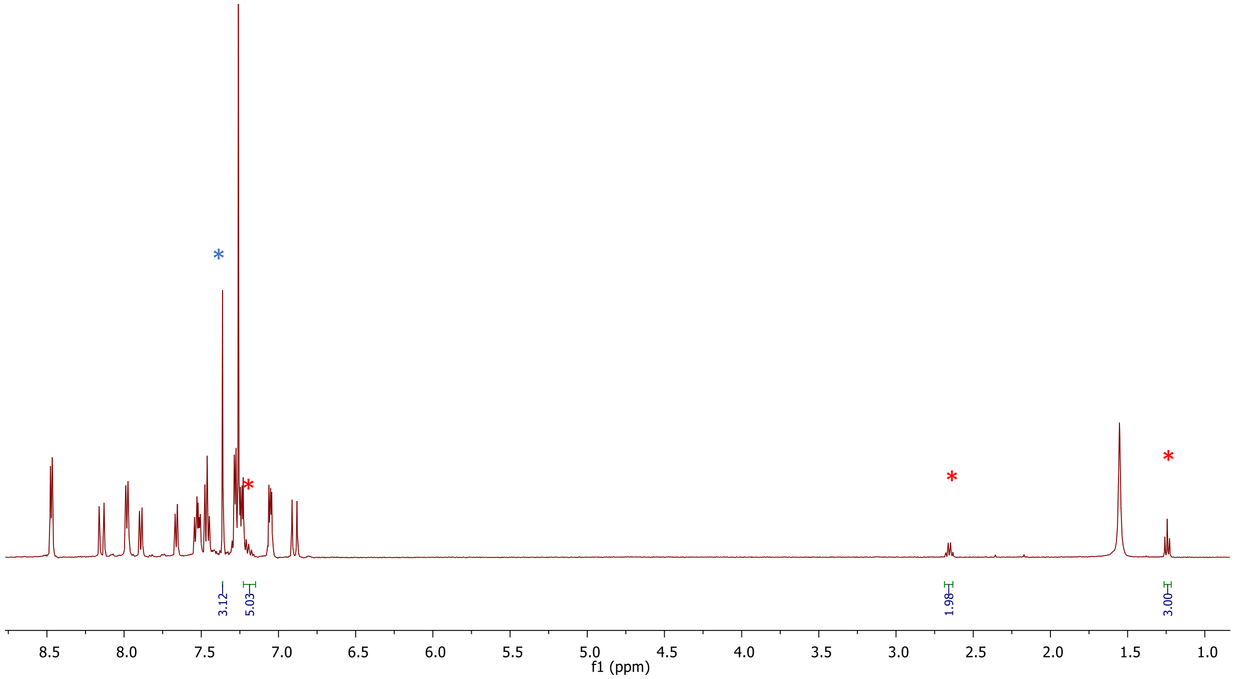

Supplementary Figure 6. ^{1}H NMR spectrum of competitive crystallization of **DBP-DEPN** between an equimolar (1:1 ratio, moles) benzene and ethylbenzene solution. Solvent inclusions: ethylbenzene (red), benzene (blue) solvent residue: chloroform (singlet,7.26 ppm).

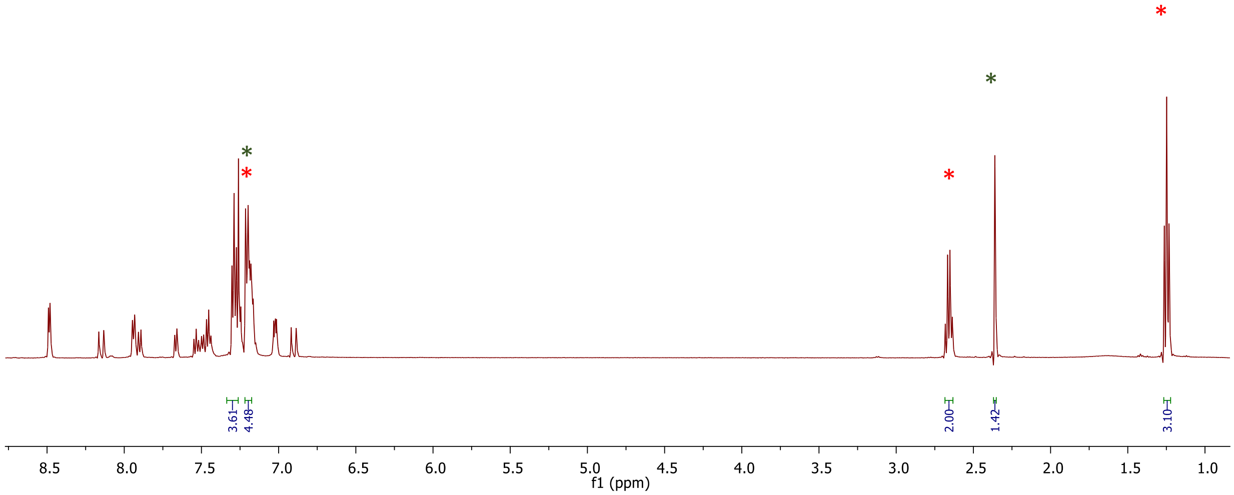

Supplementary Figure 7. ^{1}H NMR spectrum of competitive crystallization of **DBP-DEPN** between an equimolar (1:1 ratio, moles) toluene and ethylbenzene solution. Solvent inclusions: ethylbenzene (red), toluene (green) solvent residue: chloroform (singlet, 7.26 ppm).

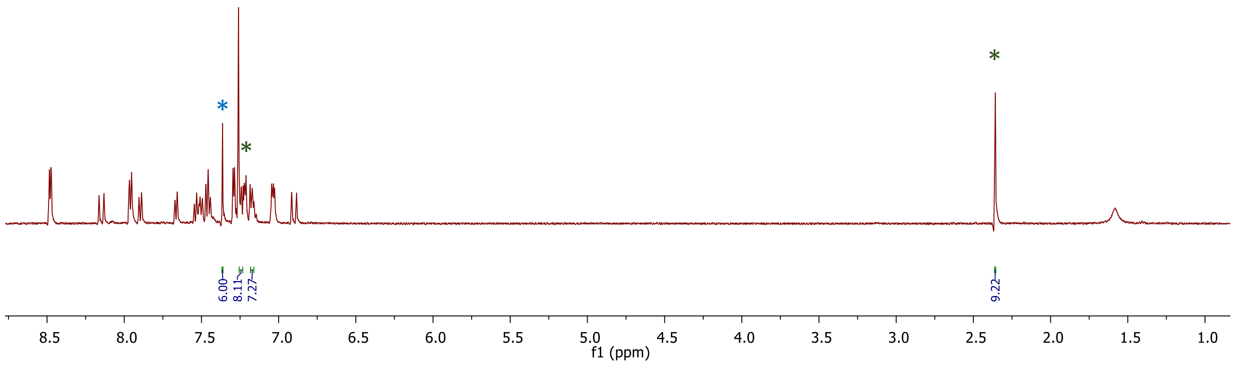

Supplementary Figure 8. ^{1}H NMR spectrum of competitive crystallization of **DBP-DEPN** between an equimolar (1:1 ratio, moles) toluene and benzene solution. Solvent inclusions: benzene (blue), toluene (green) solvent residue: chloroform (singlet, 7.26 ppm).

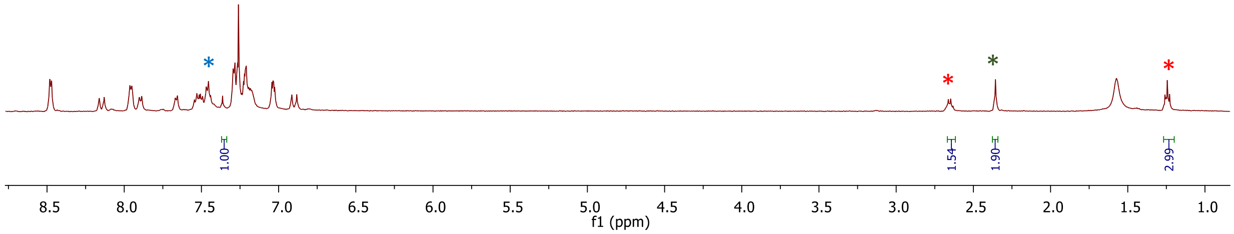

Supplementary Figure 9. ^{1}H NMR spectrum of competitive crystallization of **DBP-DEPN** between an equimolar (1:1:1 ratio, moles) ethylbenzene, toluene, and benzene solution. Solvent inclusions: benzene (blue), toluene (green) ethylbenzene (red), solvent residue: chloroform (singlet, 7.26 ppm).

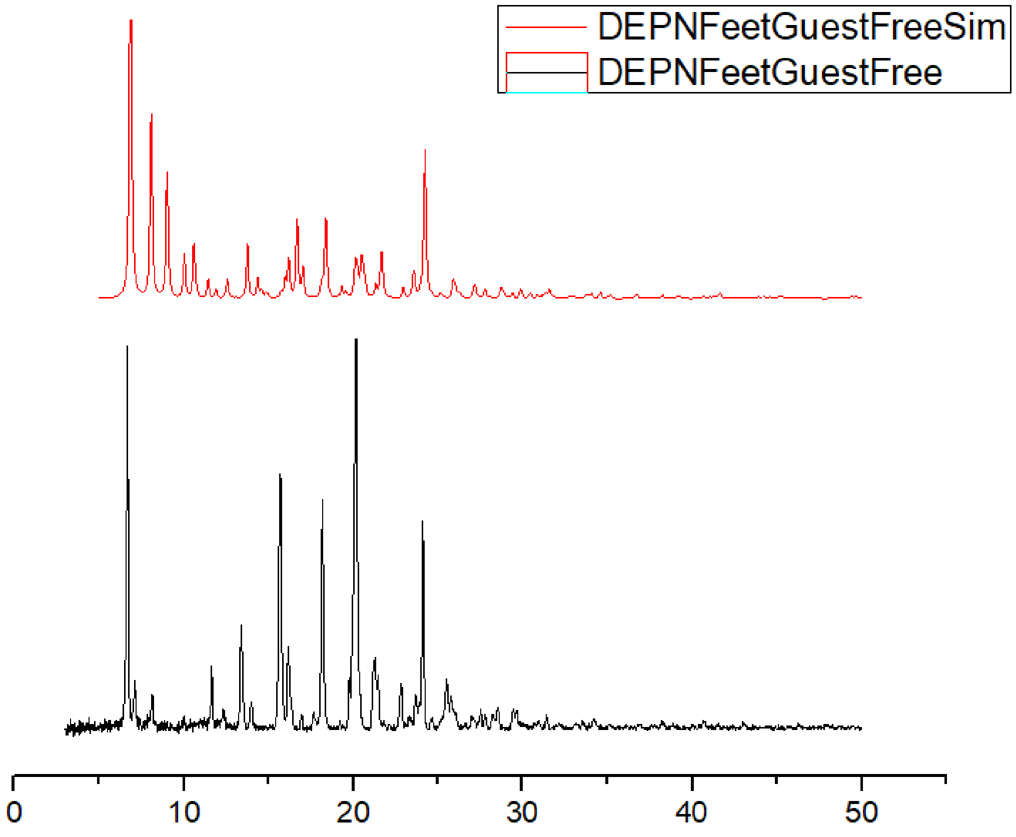

Supplementary Figure 10. Experimental and simulated PXRD patterns of **DBP-DEPN** (CH_2Cl solvate).

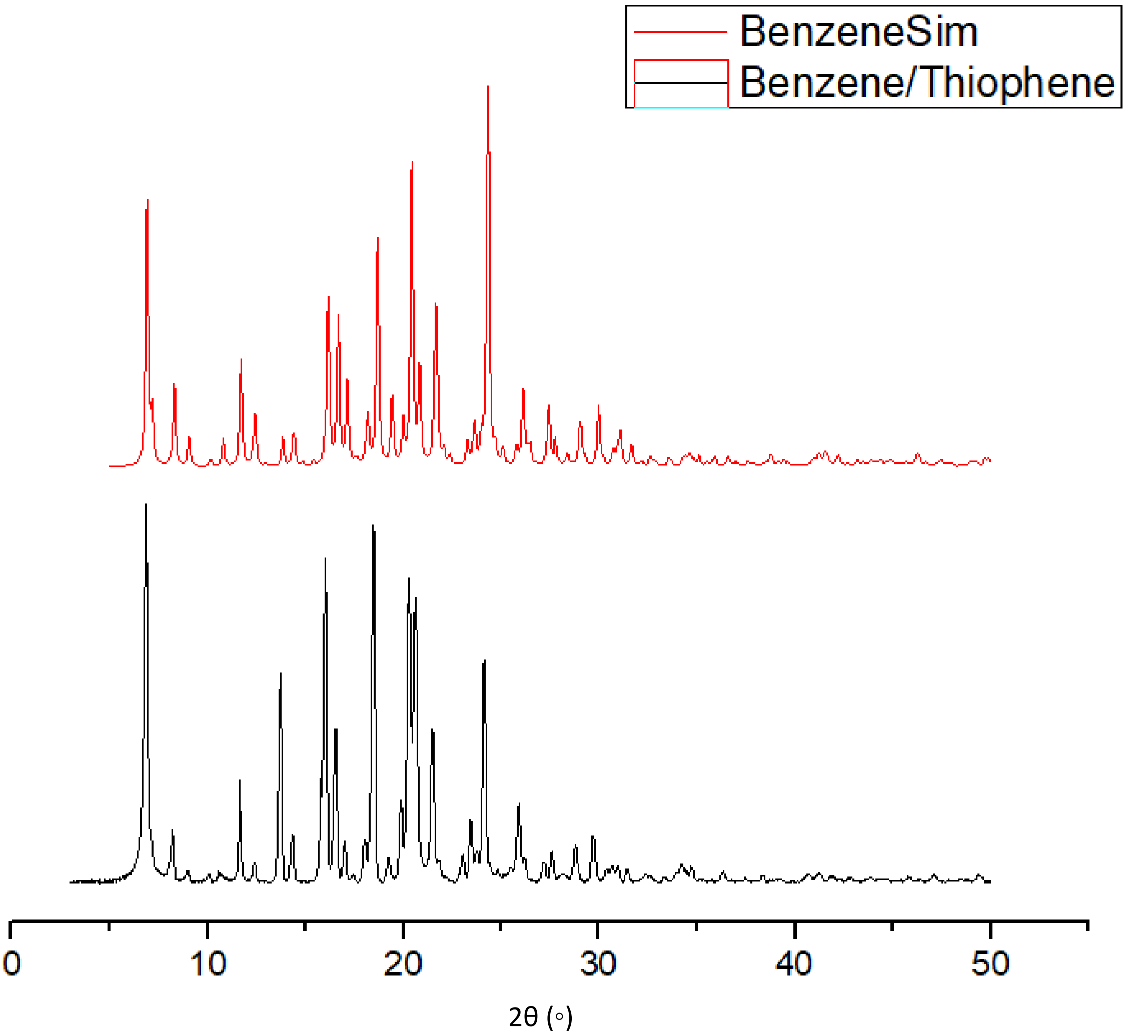

Supplementary Figure 11. Experimental and simulated PXRD patterns of **DBP-DEPN**·benzene.

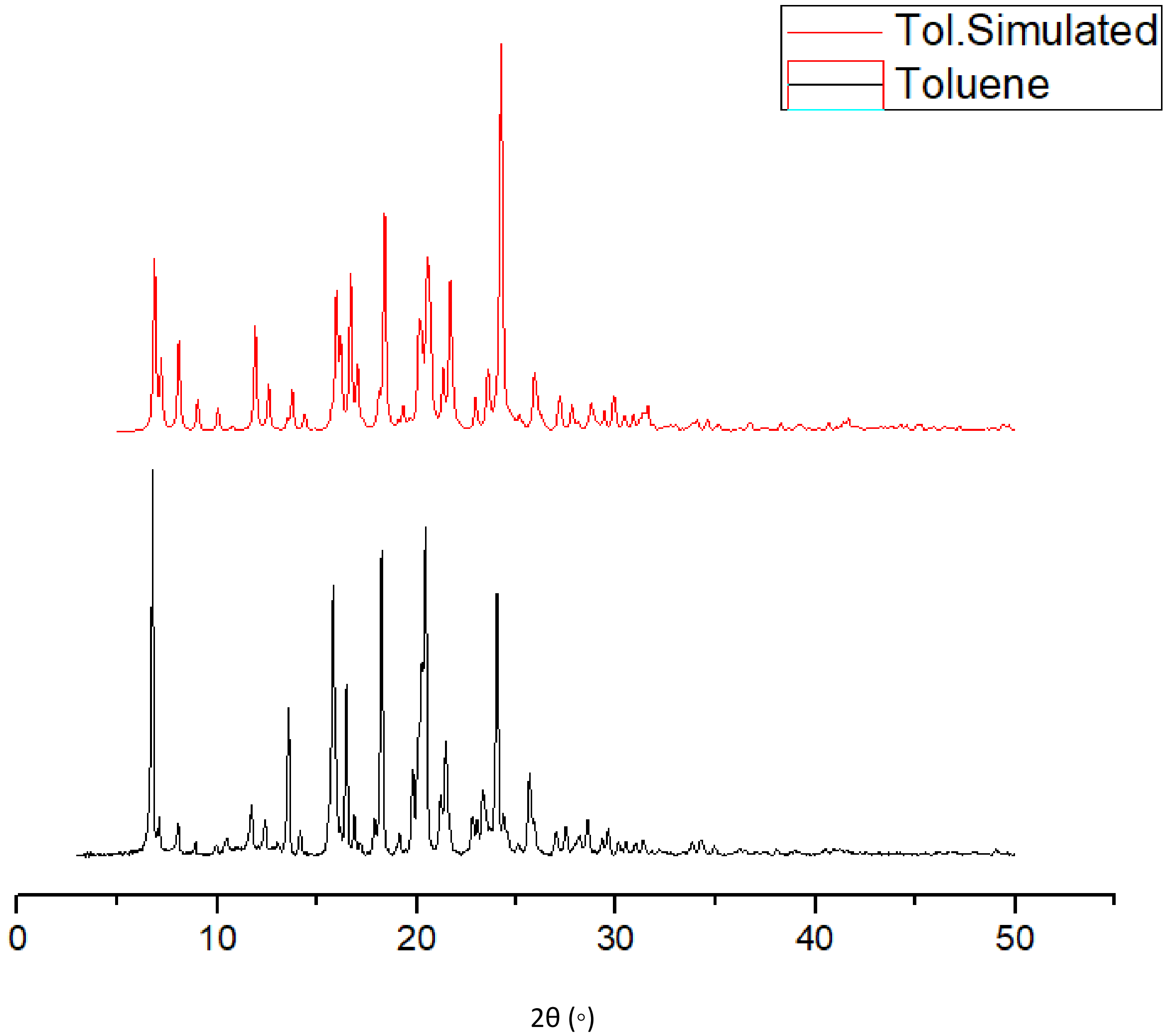

Supplementary Figure 12. Experimental and simulated PXRD patterns of **DBP-DEPN**·toluene.

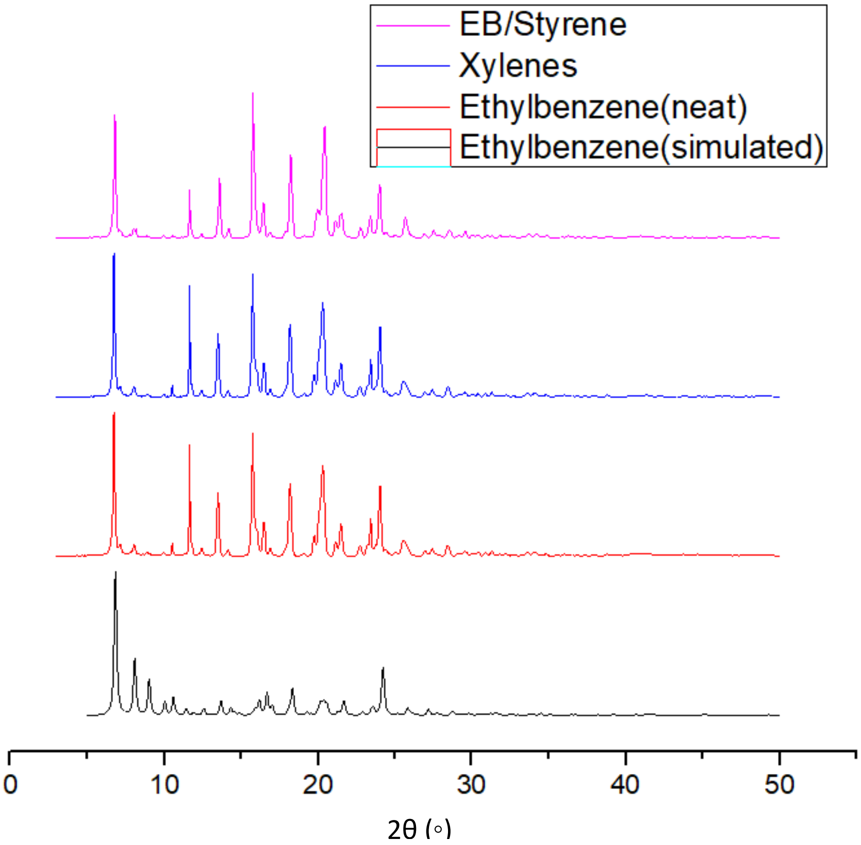

Supplementary Figure 13. Experimental and simulated PXRD patterns of **DBP-DEPN**·ethylbenzene.

Molecular Modeling

Electrostatic potential maps were generated for each **DBP-DEPN** complex by calculations at ground state in gas phase using Spartan ’18 V1.2.0 software. Calculations were performed with ωB97X-D/6-31+G* using the density functional model in a vacuum. Charge value ranges were set to a standard range of +/- 200 KJ/mole with isovalues of 0.002 for each. Single crystal lattice data was used to produce atom coordinates with hydrogens relaxed to neutron distances. Solvent inclusions removed to limit complexity.

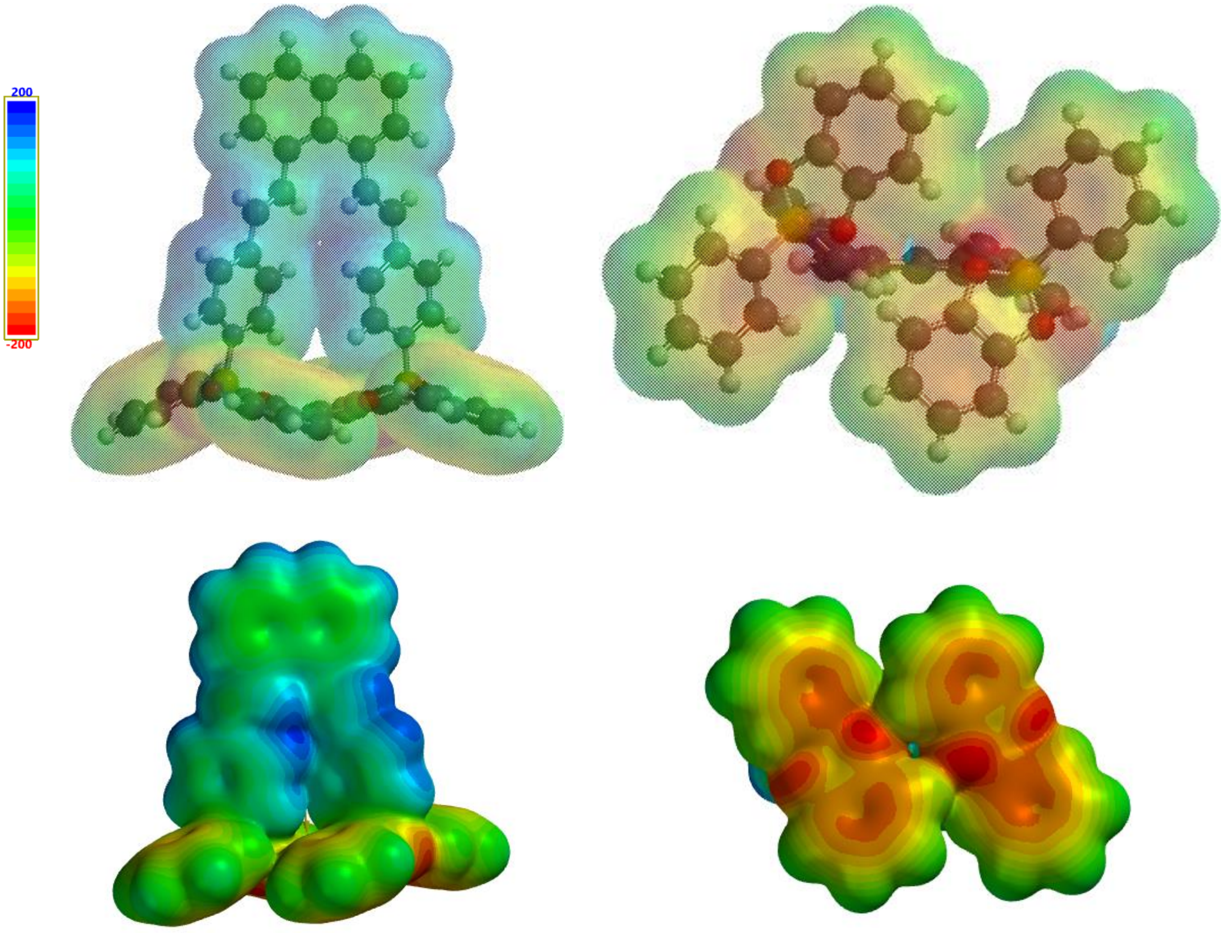

Supplementary Figure 14. Electrostatic potentials maps of **DBP-DEPN**.

Thermal measurements.

Thermogravimetric analysis data were recorded on a TA Instruments TAQ-500.

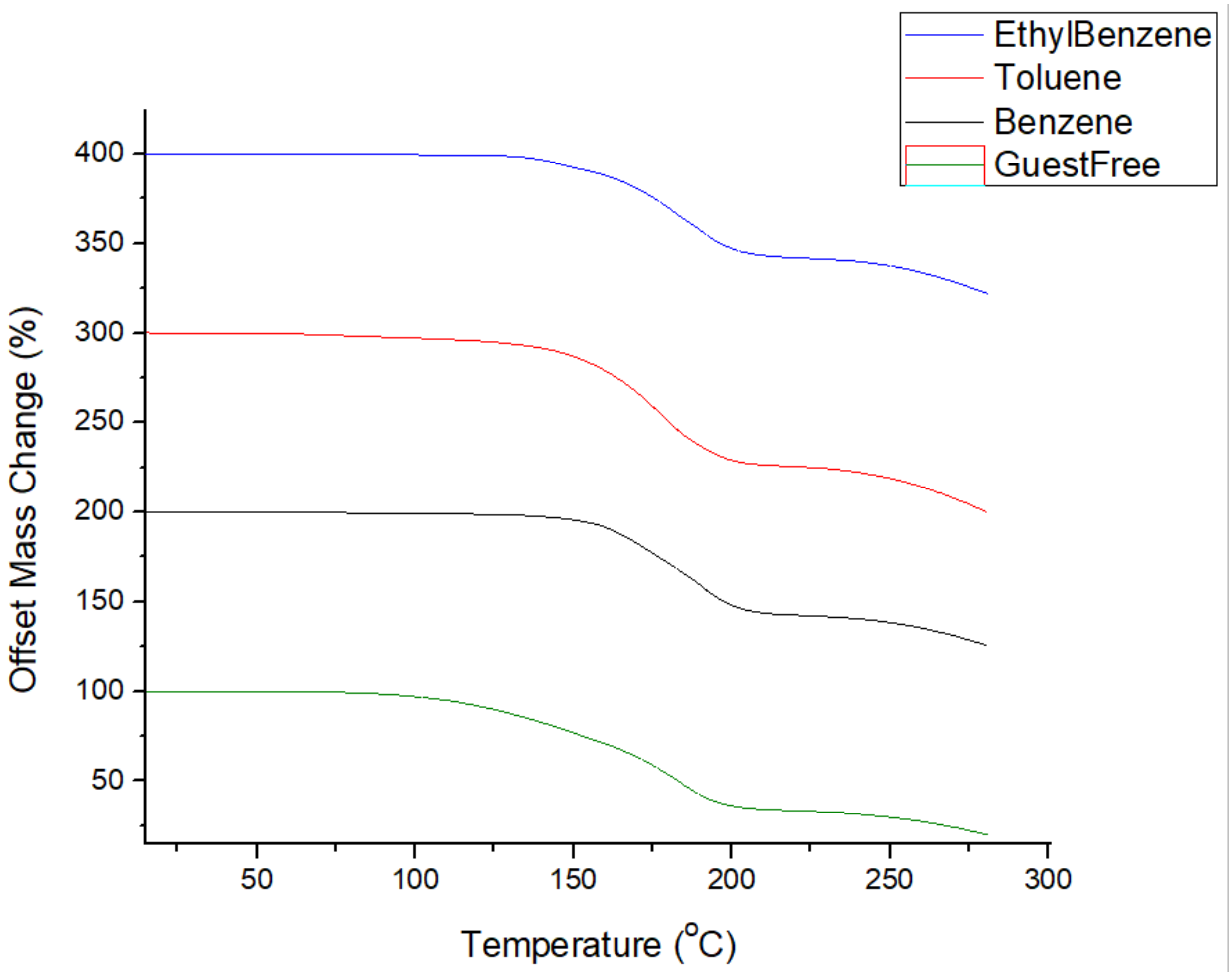

Supplementary Figure 15. TGA data of **DBP-DEPN** complexes. Ramp rate 10ºC/minute, performed after drying for 30 minutes at room temperature.

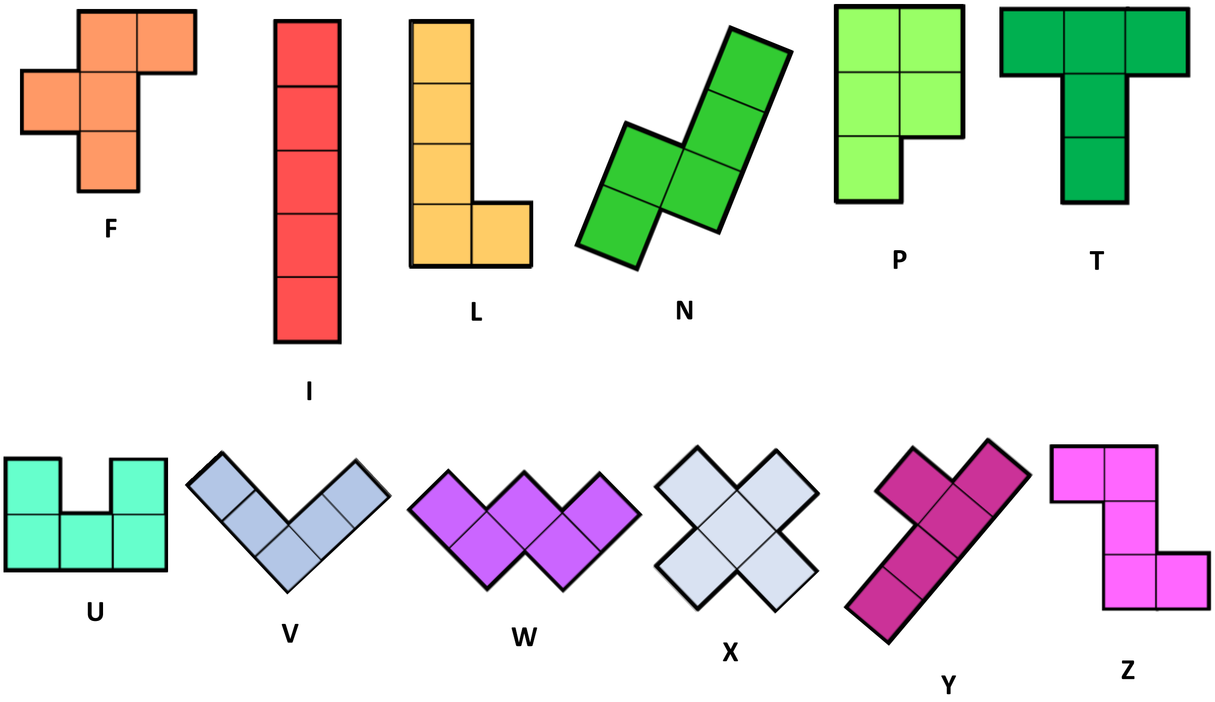

Supplementary Figure 16. The 12 possible pentominos.

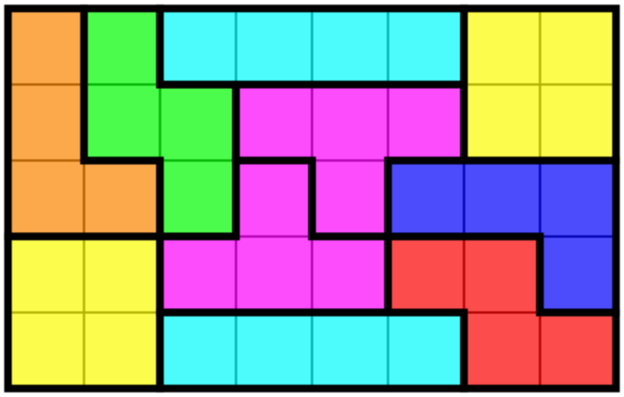

Supplementary Figure 17. Filling of a rectangle using 5-different tetrominoes.[2]

Supplementary Figure 18. The four 1-isohedral tilings for a T-pentomino.[3]

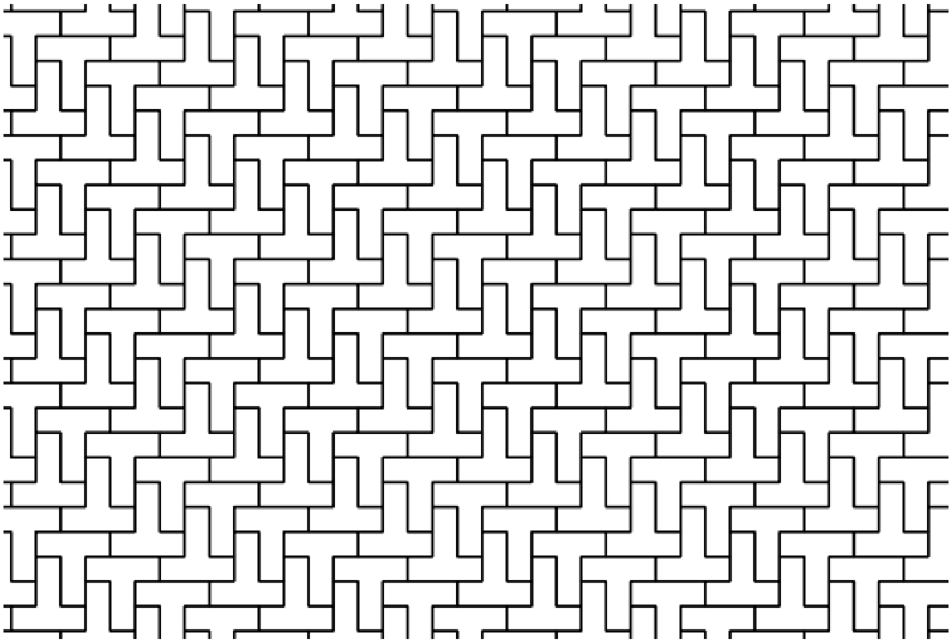

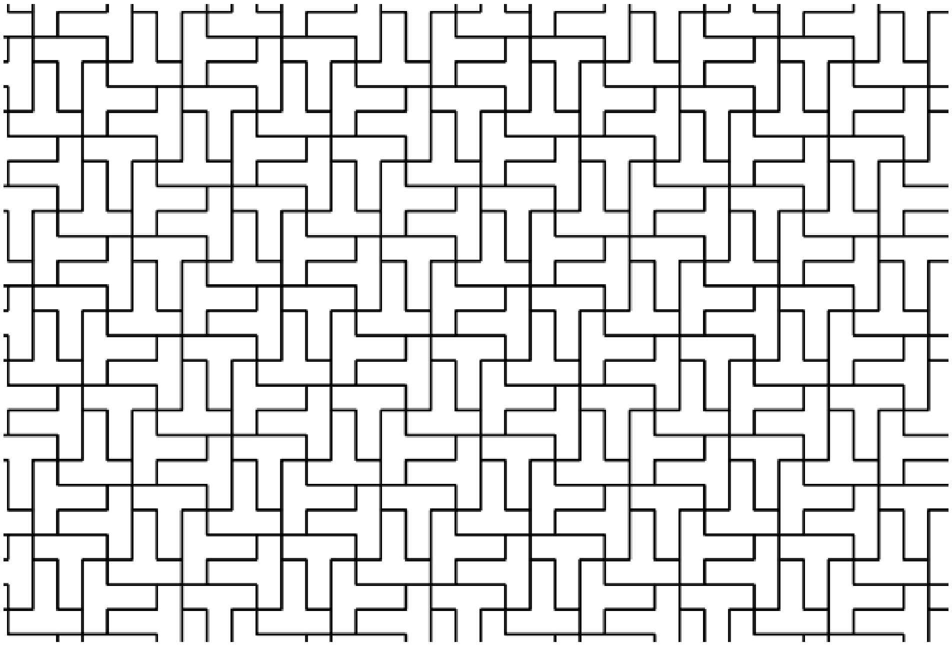

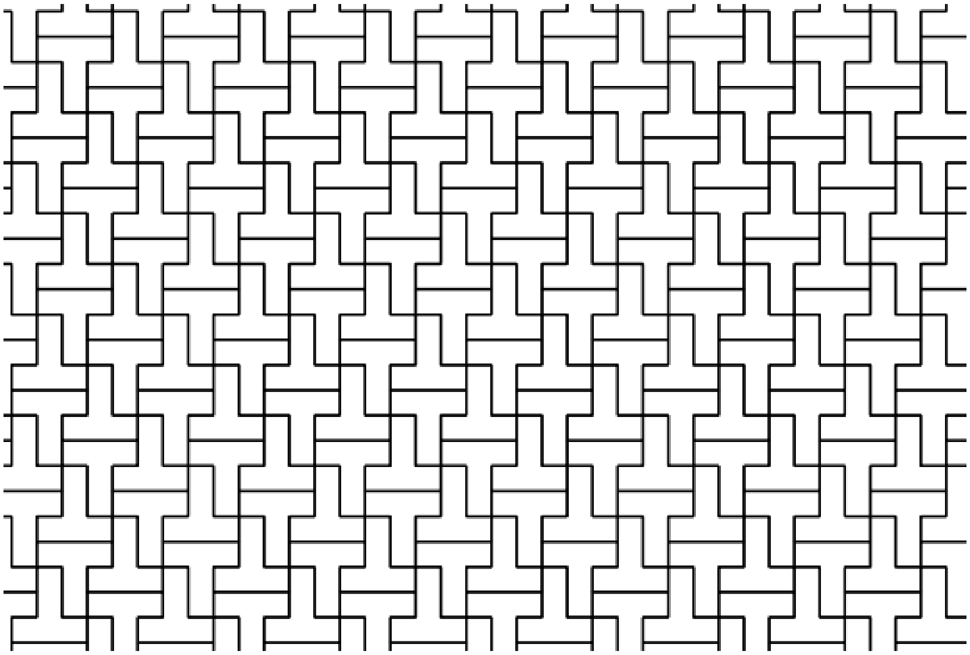

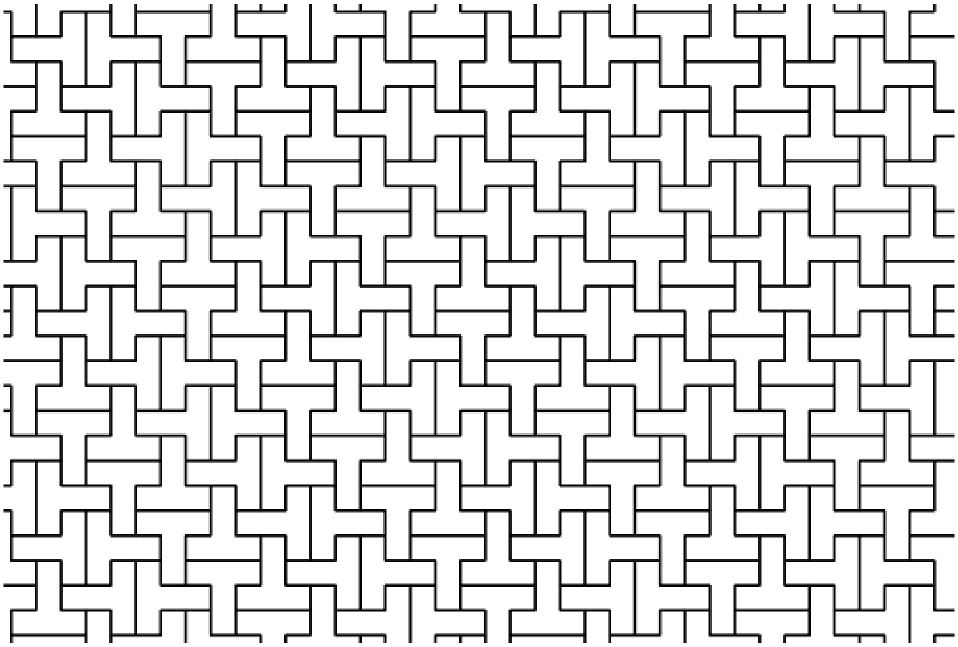

Supplementary Figure 19. The two 2-isohedral tilings for a T-pentomino.[3]

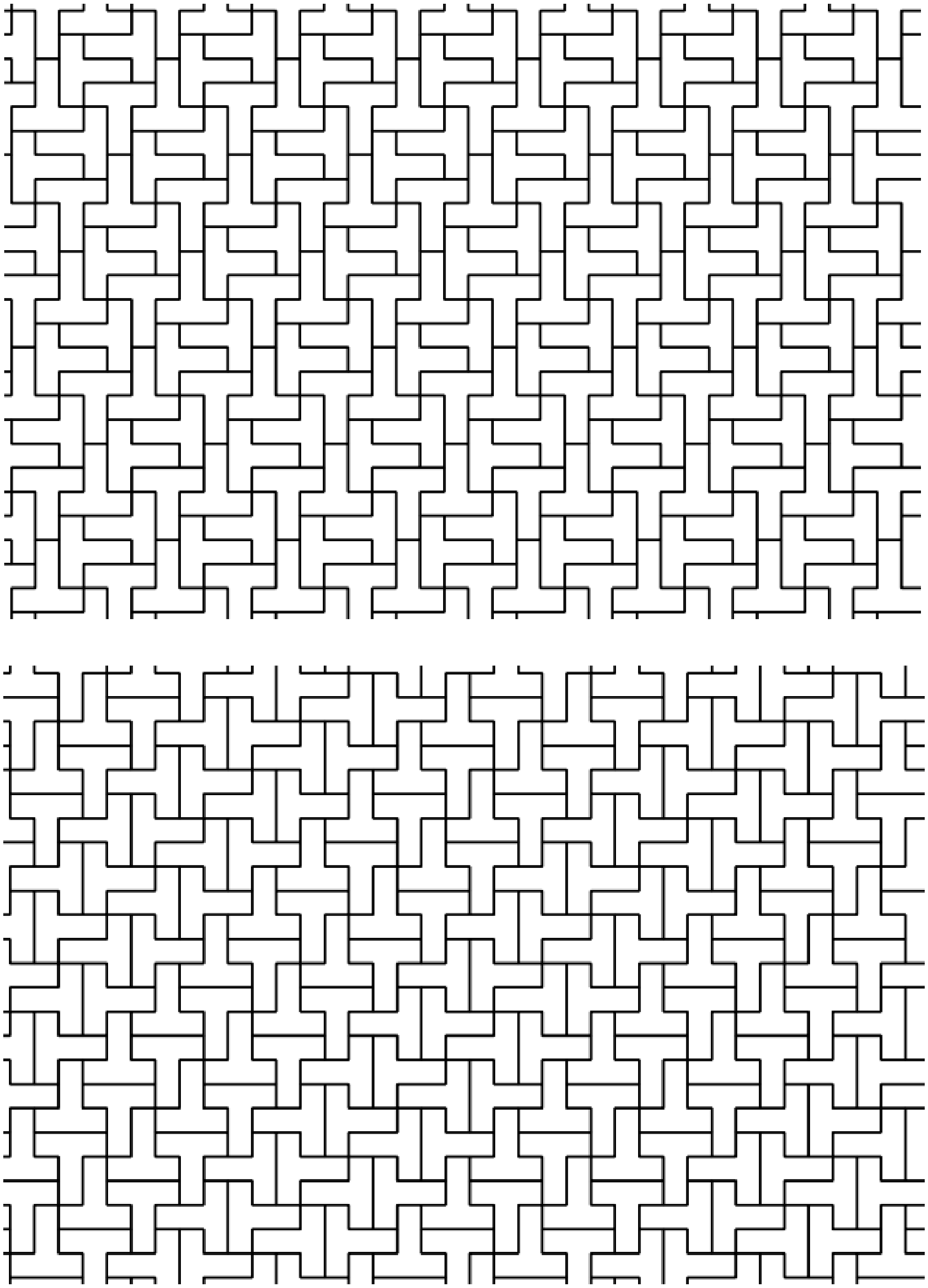

Supplementary References

1. Laird, R. C.; Sinnwell, M. A.; Nguyen, N. P.; Swenson, D. C.; Mariappan, S. V.; MacGillivray, L. R., Intramolecular [2 + 2] Photodimerization Achieved in the Solid State via Coordination-Driven Self-Assembly. *Org Lett* **2015,** *17* (13), 3233-5.
2. Brandenads, Tiling an 8×5 square with two sets of free tetrominoes. https://commons.wikimedia.org/w/index.php?curid=89396837, 2020.
3. Myers, J. Polyform tiling. (accessed June 15). https://www.polyomino.org.uk/mathematics/polyform-tiling/
